# A Promising High-Entropy Thermal Barrier Material with the Formula (Y_0.2_Dy_0.2_Ho_0.2_Er_0.2_Yb_0.2_)_3_Al_5_O_12_

**DOI:** 10.3390/ma15228079

**Published:** 2022-11-15

**Authors:** Zhanqiang Li, Junfeng Zheng, Wenjuan Zhang, Yong Zheng, Weijun Zhao, Liyan Xue, Fan Yang, Heng Chen

**Affiliations:** 1 Fujian Institute of Research on the Structure of Matter, Chinese Academy of Sciences, Fuzhou 350002, China; 2 University of Chinese Academy of Sciences, Beijing 100049, China; 3 Xiamen Key Laboratory of Rare Earth Photoelectric Functional Materials, Xiamen Institute of Rare Earth Materials, Haixi Institute, Chinese Academy of Sciences, Xiamen 361021, China; 4 Nuclear Power Institute of China, The Key Nuclear Fuel and Nuclear Materials Laboratory of China, Chengdu 610213, China; 5 Fujian Science & Technology Innovation Laboratory for Optoelectronic Information of China, Fuzhou 350108, China; 6 Fujian Province Joint Innovation Key Laboratory of Fuel and Materials in Clean Nuclear Energy System, Fujian Institute of Research on the Structure of Matter, Chinese Academy of Sciences, Fuzhou 350002, China; 7 Advanced Energy Science and Technology Guangdong Laboratory, Huizhou 516003, China

**Keywords:** rare-earth aluminates, high-entropy ceramics, thermal barrier material, chemical stability

## Abstract

YSZ has been widely used as a TBC material, but its phase change at high temperatures limits its development, thus the need for developing new thermal barrier materials resistant to high temperatures. Rare-earth aluminate ceramics with a garnet structure (Yb_3_Al_5_O_12_) have been considered as a potential thermal barrier material. The melting point of Yb_3_Al_5_O_12_ is 2000 °C, which has a potential high temperature application prospect. However, Yb_3_Al_5_O_12_ has lower thermal expansion and higher thermal conductivity than YSZ, which is a widely employed thermal barrier coating (TBC) material. To overcome these obstacles, (Y_0.2_Dy_0.2_Ho_0.2_Er_0.2_Yb_0.2_)_3_Al_5_O_12_, a high-entropy ceramic, was prepared by a solid-state reaction and pressureless sintering. The thermal conductivity of the (Y_0.2_Dy_0.2_Ho_0.2_Er_0.2_Yb_0.2_)_3_Al_5_O_12_ was 3.48 W/(m·K) at 300 K, approximately 25.48% lower than that of the Yb3Al5O12 (4.67 W/(m·K)). The thermal expansion coefficient of the (Y_0.2_Dy_0.2_Ho_0.2_Er_0.2_Yb_0.2_)_3_Al_5_O_12_ was 9.28 × 10^−6^ K^−1^ at 673-1273 K, approximately 18.52% higher than that of the Yb_3_Al_5_O_12_ (7.83 × 10^−6^ K^−1^, 673-1273 K). When the (Y_0.2_Dy_0.2_Ho_0.2_Er_0.2_Yb_0.2_)_3_Al_5_O_12_ was annealed at 1550 °C for 7 days, its average grain size only increased from 0.7 μm to 1.3 μm. Moreover, the (Y_0.2_Dy_0.2_Ho_0.2_Er_0.2_Yb_0.2_)_3_Al_5_O_12_ exhibited better chemical stability and a lower grain growth rate than the Yb_3_Al_5_O_12_. This study reveals that (Y_0.2_Dy_0.2_Ho_0.2_Er_0.2_Yb_0.2_)_3_Al_5_O_12_ is a promising candidate for the future generation of thermal barrier materials.

## 1. Introduction

Thermal barrier coatings (TBCs) are widely employed in gas turbine engines for aerospace, power generation, and marine applications and can protect the superalloy by reducing the alloy surface temperature and increasing the engine efficiency of gas turbines [1,2,3,4,5,6]. TBCs must be able to withstand extreme temperature cycling and thermal shock [7,8,9]. Therefore, the selection of TBCs is restricted by some basic requirements: (1) excellent high-temperature stability, (2) a thermal expansion coefficient (TEC) consistent with the metallic substrate, (3) a low grain growth rate, (4) low thermal conductivity, and (5) chemical inertness. To date, YSZ has been widely employed as a TBC material, but the limitations of phase transformation at a high temperature and high oxygen diffusivity make it unsuitable for the high operating temperature of gas turbine engines [10,11]. At high temperatures, the non-equilibrium tetragonal phase in YSZ is prone to decomposition to generate t-phase and c-phase, and the cumulative effect of the volume expansion due to the phase change will cause cracks in the coating. Moreover, oxygen ions from YSZ above 800 °C tend to oxidize the metal substrate through the coating [12,13,14]. Therefore, YSZ cannot serve for a long time at temperatures above 1200 °C. Thus, novel TBC materials with better high-temperature stability are urgently needed.

The physicochemical properties of rare-earth elements have made them a research priority in the field of thermal barrier coatings [15,16,17]. Wang et al. reported that the thermal barrier coating of La_1.4_Nd_0.6_Zr_2_O_7_(LNZ) on a Mo substrate was prepared by air plasma spraying with a self-developed LNZ thermal spray powder [18]. Xu et al. reported that (Sm_0.2_La_0.8_)_2_(Zr_0.7_Ce_0.3_)_2_O_7_ (SmLZC), as a candidate material for novel thermal barrier coatings, was prepared by electron beam-physical vapor deposition (EB-PVD) [19]. Thus, rare-earth oxides exhibit good feasibility in the field of thermal barrier coatings. Therein, Yb_3_Al_5_O_12_ exhibits excellent high-temperature stability, isotropic elastic properties, and low intrinsic thermal conductivity and has recently been recognized as a potential thermal barrier material [20,21,22,23]. The melting point of Yb_3_Al_5_O_12_ is 2000 °C, which has a potential high-temperature application prospect. Moreover, theoretical results obtained by Klemm confirmed that Yb_3_Al_5_O_12_ has better chemical stability than several other promising TBCs, such as Y_2_Si_2_O_7_ and Yb_2_Si_2_O_7_ [24]. However, Yb_3_Al_5_O_12_ has lower expansion and higher thermal conductivity than YSZ, which is a widely used TBC material [22]. The linear thermal expansion of Yb_3_Al_5_O_12_ was in the range from 298 to 1273 K, 7.83 × 10^−6^ K^−1^, which was lower than that of YSZ ((10-11) × 10^−6^ K^−1^). The measured thermal conductivities of Yb_3_Al_5_O_12_ at 300 and 1400 K are 4.67 W/(m·K) and 2.05 W/(m·K), which were greater than those of YSZ. Thus, decreasing the thermal conductivity of Yb_3_Al_5_O_12_ and increasing the coefficient of thermal expansion of Yb_3_Al_5_O_12_ are critical to expanding its applications.

Currently, the emergence of high-entropy ceramics (HECs) has attracted attention [25,26,27,28,29,30]. It has been reported that HECs efficiently enhance the thermal performance of thermal barrier materials because they have higher temperature stability, more consistent TECs, and a slower grain growth rate than single-component compounds. Lattice distortion and component disorder can increase phonon scattering and thus reduce thermal conductivity. In addition, better chemical stability can be achieved due to retarded diffusion. Some researchers have successfully synthesized different kinds of high-entropy oxides (HEOs) for TBC applications. An increase of 6 h in annealing results in only a 1 μm increase in grain size for (5RE_0.2_)Ta_3_O_9_, while this increases to approximately 3 μm for EuTa_3_O_9_ [31]. Sun et al. reported that the thermal conductivities of (Eu_0.2_Er_0.2_Lu_0.2_Y_0.2_Yb_0.2_)_2_O_3_ and (Sm_0.2_Er_0.2_Lu_0.2_Y_0.2_Yb_0.2_)_2_O_3_ are 5.10 W/(m·K) and 4.60 W/(m·K), which are 23.8% and 21.5% of that of Y_2_O_3_ (21.40 W/(m·K)), respectively [32]. Ren et al. prepared (Y_1/4_Ho_1/4_Er_1/4_Yb_1/4_)_2_SiO_5_ silicate as a multifunctional TBC material, and it showed good resistance to high-temperature water vapor [33]. In addition, due to the excellent properties of HECs, Chen et al. reported a high-entropy rare-earth aluminate (Y_0.2_Yb_0.2_Lu_0.2_Eu_0.2_Er_0.2_)_3_Al_5_O_12_ [34]. Thus, HECs can effectively enhance the combined properties of thermal barrier materials.

Herein, we design and synthesize a promising high-entropy rare-earth aluminate ceramic with garnet, (Y_0.2_Dy_0.2_Ho_0.2_Er_0.2_Yb_0.2_)_3_Al_5_O_12_, by the solid-state reaction method. The standards for the component design are as follows: (1) to guarantee the formation of solid solutions, differences in the size of the rare-earth ions must not be greater than 15.0%; (2) each component must have the structure of garnet; and (3) no phase change may occur at the working temperature. In addition, in terms of the selected elements, we try to use low-cost rare-earth elements to further reduce the economic problem. The thermal expansion coefficient, thermal conductivity, high-temperature stability, chemical inertness and hardness of (Y_0.2_Dy_0.2_Ho_0.2_Er_0.2_Yb_0.2_)_3_Al_5_O_12_ are studied in the present work. These properties are valuable and beneficial to the potential application of (Y_0.2_Dy_0.2_Ho_0.2_Er_0.2_Yb_0.2_)_3_Al_5_O_12_ as the future generation of thermal barrier materials.

## 2. Experimental Section

### 2.1. Synthesis of (Y_0.2_Dy_0.2_Ho_0.2_Er_0.2_Yb_0.2_)_3_Al_5_O_12_

**Figure 1:**
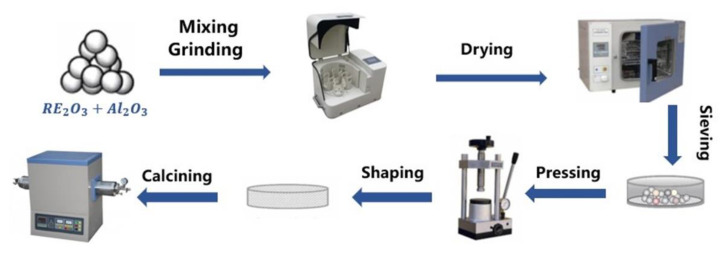
Schematic illustration of the preparation process of (Y_0.2_Dy_0.2_Ho_0.2_Er_0.2_Yb_0.2_)_3_Al_5_O_12_ by the solid-state reaction method and the pressureless sintering method.

Y_2_O_3_ (99.99%), Dy_2_O_3_ (99.99%), Ho_2_O_3_ (99.99%), Er_2_O_3_ (99.99%), Yb_2_O_3_ (99.99%), and Al_2_O_3_ (99.999%) were purchased from Aladdin Reagent Ltd., Co. (Shanghai, China). Figure 1 shows the synthesis of (Y_0.2_Dy_0.2_Ho_0.2_Er_0.2_Yb_0.2_)_3_Al_5_O_12_ via a solid-state reaction. The total molar ratio of rare-earth elements to Al was 3:5. First, five rare-earth oxides with equal molar ratios were mixed with Al_2_O_3_ and alcohol, and ground at 360 r/min for 10 h using a planetary ball mill. Afterward, the obtained solution was dried in an oven at 80 °C for 10 h. The obtained powder mixture was pressed and molded at 5 MPa. Afterwards, the (Y_0.2_Dy_0.2_Ho_0.2_Er_0.2_Yb_0.2_)_3_Al_5_O_12_ powders were synthesized by sintering at 1600 °C for 2 h. The as-prepared powder was ball-milled for 10 h and then dried and sieved through a 400 mesh sieve to obtain fine particles. The samples were densified by the pressureless sintering method. The process of cold isostatic pressing is that the powder is pressed through a mold, then pressed again in a cold isostatic press at a pressure of 200 MPa to make itself more dense, and then calcined in a muffle furnace at a temperature of 1650 °C for 5 h. Finally, the densified bulk samples can be obtained.

### 2.2. Characterization of the Materials

A scanning speed of 2°/min was used for X-ray diffraction (XRD, Miniflex 600 instrument, Tokyo, Japan) with Cu Kα radiation to determine the phase structure of the powder samples. Scanning electron microscopy (SEM, Apreo S LoVac Thermo Fisher Scientific, Waltham, MA, USA) was combined with field-emission transmission electron microscopy (TEM, FEI/Talos F200X G2, Lincoln, NE, USA) and energy-dispersive spectrometry (EDS) to examine the morphology and chemical composition of the samples. The TEC of the bulk samples was determined by a thermal expansion meter (Cryoall C15V, Beijing, China) with a size of Φ 30.0 mm × 2.0 mm. The thermal conductivity of the bulk samples was examined by a thermal constant analyzer (TPS 2500, Hot Disk, Göteborg, Sweden) with a size of Φ 6.0 mm × 10.0 mm. The thermal conductivities of the dense materials were corrected by the following Formula (1) [35].

(1) $$\frac{K_{S}}{K}=1-1.5P$$

where *K_S_* is the thermal conductivity obtained by the test, *K* is the thermal conductivity of the fully dense materials, and *P* is the porosity.

A Vickers hardness test was conducted using a micro hardness tester (FT FM-700, Tokyo, Japan). The samples for Vickers hardness testing were cut by a diamond saw from the as-prepared bulk material and then polished down to 1.0-μm diamond grits. The Vickers hardness number was determined by a microhardness tester with loads of 1, 3, 5, and 10 N, respectively. Loads of 1, 3, 5, and 10 N were applied with a dwell time of 15 s. The Vickers hardness tests were calculated by using Formula (2).

(2) $$Hv=P/S=2P\sin\left(\frac{\theta }{2}\right)/d^{2}=1854.4P/d^{2}$$

where *P* is the load, and *d* is the diagonal length of indentation. Five measurements for each load were taken to obtain the average value of hardness.

The cell dimension was calculated by the Rietveld refinement using GSAS software (EXPGUI-GSAS), and the reliability factors of *R_p_* and *R_wp_* were calculated by using Formulas (3) and (4) [36].

(3) $$R_{p}=\sum |Y_{oi}-Y_{ci}|/\sum Y_{oi}$$

(4) $$R_{wp}={\left[W_{i}{\left(Y_{oi}-Y_{ci}\right)}^{2}/W_{i}Y^{2}{}_{oi}\right]}^{1/2}$$

The Archimedes method was used to measure the bulk density (*ρ*), while Formula (5) was used to calculate the theoretical density.

(5) $$\rho_{th}=m/v$$

where *m* is the mass of a unit cell of HE-RE_3_Al_5_O_12_ and *V* is the cell volume. The relative density was calculated from the ratio of the actual density to the theoretical density (*ρ*/*ρ_th_*).

The concentrations of dissolved ions in the leachates were determined by inductivity-coupled plasma mass spectrometry (ULTIMA 2, Horiba Jobin Yvon S.A.S, Edison Township, NJ, USA). A leaching test of the bulk samples by atmospheric pressure sintering at 1640 °C for 5 h was performed in a nitric acid solution in PTFE for 1–5 d. The HE-RE_3_Al_5_O_12_ ceramics, which had a diameter of 15 mm and a height of 2 mm, were polished, cleaned, and dried. The ratio of the geometric surface area of the sample to the volume of the leachate was maintained at 1:10 cm^−1^. The normalized element mass leaching rates (*LR_i_*) of the (Al) were obtained based on Formula (6) [37].

(6) $$LR_{i}=C_{i}\cdot V/f_{i}\cdot S\cdot t$$

where *C_i_* is the concentration of elements (Al) in the leaching solution, *V* is the volume of the leaching solution, *f_i_* is the proportion of element I in the ceramic, *S* is the surface area of the ceramic, and *t* is the date of leaching.

## 3. Results and Discussion

**Figure 2:**
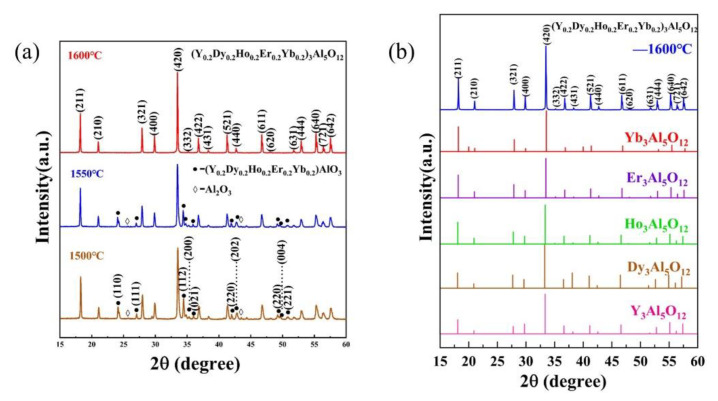
(**a**) XRD patterns of (Y_0.2_Dy_0.2_Ho_0.2_Er_0.2_Yb_0.2_)_3_Al_5_O_12_ at different temperatures (1500 °C, 1550 °C, 1600 °C); (**b**) XRD patterns of (Y_0.2_Dy_0.2_Ho_0.2_Er_0.2_Yb_0.2_)_3_Al_5_O_12_ at 1600 °C for 2 h with those of single-component RE_3_Al_5_O_12_ (RE = Y, Dy, Ho, Er, Yb) obtained from JCPDF cards.

**Figure 3:**
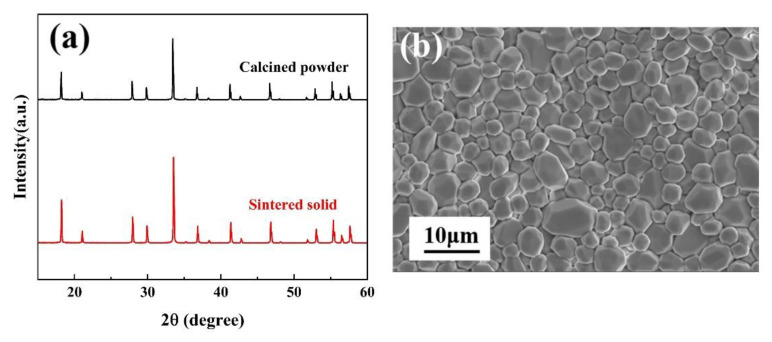
(**a**) XRD patterns of calcined powder and the sintered solid; (**b**) surface SEM image of (Y_0.2_Dy_0.2_Ho_0.2_Er_0.2_Yb_0.2_)_3_Al_5_O_12_.

Figure 2a shows the XRD patterns of (Y_0.2_Dy_0.2_Ho_0.2_Er_0.2_Yb_0.2_)_3_Al_5_O_12_ at different temperatures (1500 °C, 1550 °C, 1600 °C). As can be seen from Figure 2a, when the synthesis temperature is 1500 °C or 1550 °C, impurity phases are (Y_0.2_Dy_0.2_Ho_0.2_Er_0.2_Yb_0.2_)AlO_3_ and Al_2_O_3_. Figure 2b shows the XRD patterns of (Y_0.2_Dy_0.2_Ho_0.2_Er_0.2_Yb_0.2_)_3_Al_5_O_12_ at 1600 °C for 2 h, with those of the single-component RE_3_Al_5_O_12_ (RE = Y, Dy, Ho, Er, Yb) obtained from JCPDF cards. As can be seen from Figure 2b, when the green bodies were heated to 1600 °C in ai with a dwell time of 2 h, no other impurity peak phase is observed. The XRD patterns of the (Y_0.2_Dy_0.2_Ho_0.2_Er_0.2_Yb_0.2_)_3_Al_5_O_12_ powder matched those of a single component, indicating that the (Y_0.2_Dy_0.2_Ho_0.2_Er_0.2_Yb_0.2_)_3_Al_5_O_12_ synthesized in this study had the same structure as a single component. No other contaminants were indicated by the diffraction peaks, confirming that a solid solution was prepared. Thus, a pure-phase (Y_0.2_Dy_0.2_Ho_0.2_Er_0.2_Yb_0.2_)_3_Al_5_O_12_ solid solution was successfully prepared. Figure 3a shows the XRD patterns of the calcined powder and the sintered solid. The XRD patterns were consistent before and after calcination, indicating that no impurities were introduced. The absence of holes and cracking can be seen in the SEM image in Figure 3b, indicating that the sintered (Y_0.2_Dy_0.2_Ho_0.2_Er_0.2_Yb_0.2_)_3_Al_5_O_12_ exhibited a high relative density.

**Figure 4:**
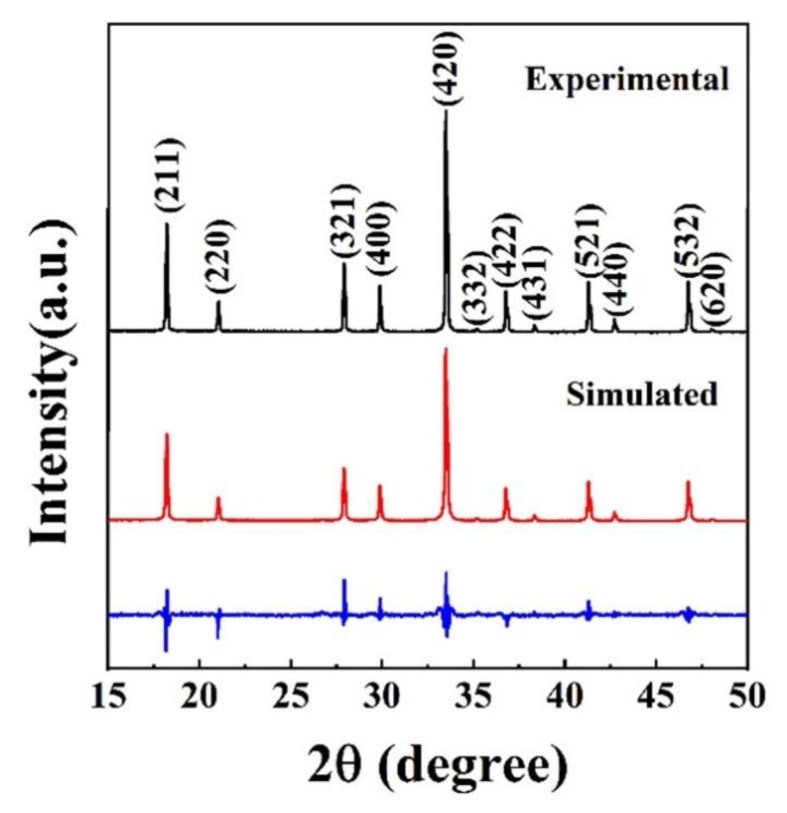
XRD patterns of the as-synthesized (Y_0.2_Dy_0.2_Ho_0.2_Er_0.2_Yb_0.2_)_3_Al_5_O_12_ powders, together with the Rietveld refinement data.

As shown in Figure 4, the cell dimension of the (Y_0.2_Dy_0.2_Ho_0.2_Er_0.2_Yb_0.2_)_3_Al_5_O_12_ was calculated by the Rietveld refinement using GSAS software, and the reliable factors calculated via the equations are *R_p_* = 3.38% and *R_wp_* = 4.41%. The refinement results are generally considered reliable for values of *R_p_* and *R_wp_* below 10.00%. The (Y_0.2_Dy_0.2_Ho_0.2_Er_0.2_Yb_0.2_)_3_Al_5_O_12_ ceramic lattice parameters were found to be *a* = *b* = *c* = 11.992 Å, which is approximately equal to the average cell size of the five single components (11.986 Å). Table 1 shows the refined cell dimensions and theoretical density of (Y_0.2_Dy_0.2_Ho_0.2_Er_0.2_Yb_0.2_)_3_Al_5_O_12_ and the five single-component garnets obtained from jade. The theoretical density of (Y_0.2_Dy_0.2_Ho_0.2_Er_0.2_Yb_0.2_)_3_Al_5_O_12_ was 6.02 g/cm^3^, based on the refined cell parameters. The sintered density, calculated by Archimedes’ approach, is 5.88 g/cm^3^, 97.67% of the theoretical value (6.02 g/cm^3^).

**Figure 5:**
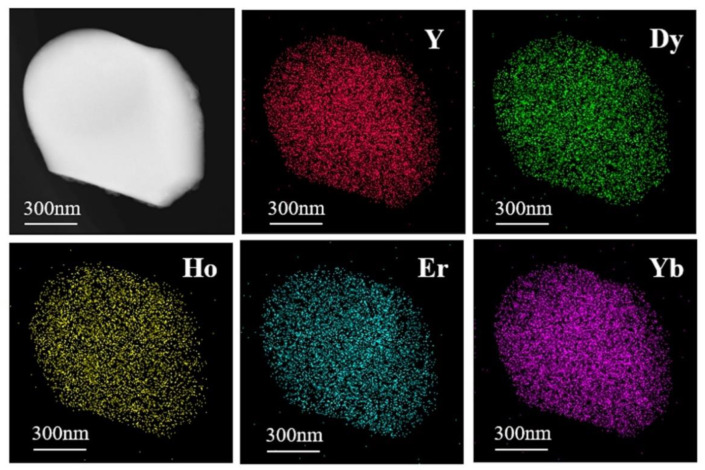
Elemental compositions of Y, Dy, Ho, Er, Yb in HAADF images.

**Figure 6:**
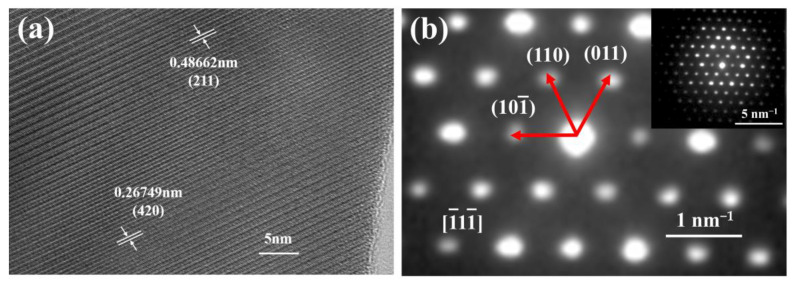
The power of (**a**) the high-resolution TEM image and (**b**) the corresponding SAED pattern.

Figure 5 shows the high-angle annular dark-field (HAADF) image and element mapping of the (Y_0.2_Dy_0.2_Ho_0.2_Er_0.2_Yb_0.2_)_3_Al_5_O_12_ powders. The distribution of elements is clearly uniform, demonstrating the uniformity of the solid solution. The XRD and TEM–EDS results suggest that single-phase aluminates with excellent chemical uniformity were synthesized by the solid-state reaction approach. Figure 6a shows that the (Y_0.2_Dy_0.2_Ho_0.2_Er_0.2_Yb_0.2_)_3_Al_5_O_12_ ceramics were further characterized by HR-TEM. Two positions were selected to calculate the lattice fringes, which exhibited typical (211) and (420) lattice planes with D-spacings of 0.488 nm and 0.267 nm, respectively. These results are consistent with the broad XRD peaks of the (Y_0.2_Dy_0.2_Ho_0.2_Er_0.2_Yb_0.2_)_3_Al_5_O_12_ at 18.2° (0.48665 nm, (211)) and 33.5° (0.26662 nm, (420)). The selected area electron diffraction (SAED) pattern of the (Y_0.2_Dy_0.2_Ho_0.2_Er_0.2_Yb_0.2_)_3_Al_5_O_12_ powders (Figure 6b) shows that (Y_0.2_Dy_0.2_Ho_0.2_Er_0.2_Yb_0.2_)_3_Al_5_O_12_ has a single-crystal ordered garnet structure.

Thermal conductivity is essential for assessing the thermal insulation properties of insulating materials. Table 2 shows the thermal conductivity of the (Y_0.2_Dy_0.2_Ho_0.2_Er_0.2_Yb_0.2_)_3_Al_5_O_12_, (Y_0.2_Yb_0.2_Lu_0.2_Eu_0.2_Er_0.2_)_3_Al_5_O_12_, and Y_3_Al_5_O_12_ at 300 K. The thermal conductivity of the (Y_0.2_Dy_0.2_Ho_0.2_Er_0.2_Yb_0.2_)_3_Al_5_O_12_ ceramics was found to be 3.48 W/(m·K) at 300 K, approximately 25.48% and 8.66% lower than that of Yb_3_Al_5_O_12_ (4.67 W/(m·K)) and (Y_0.2_Yb_0.2_Lu_0.2_Eu_0.2_Er_0.2_)_3_Al_5_O_12_ (3.81 W/(m·K)), indicating that (Y_0.2_Dy_0.2_Ho_0.2_Er_0.2_Yb_0.2_)_3_Al_5_O_12_ is a potential thermal barrier material [32]. The thermal conductivities of rare-earth aluminates are slightly higher than commercial TBC material YSZ, which is 2.50–3.20 W/(m·K) [38]. Five equimolar rare-earth cations are present in (Y_0.2_Dy_0.2_Ho_0.2_Er_0.2_Yb_0.2_)_3_Al_5_O_12_ at crystallographic sites randomly occupying the same lattice. The mismatch between the masses and radii of these cations leads to large lattice distortions and intense phonon scattering in the material. Among the four core effects of high-entropy materials is lattice distortion, which may play a significant role in the decrease in the thermal conductivity of garnets [39].

**Figure 7:**
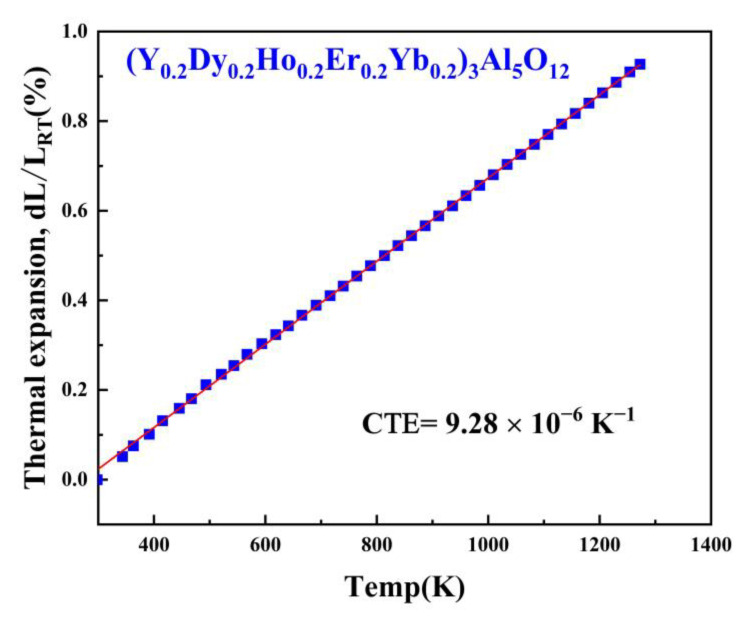
The linear thermal expansion coefficient of polycrystalline (Y_0.2_Dy_0.2_Ho_0.2_Er_0.2_Yb_0.2_)_3_Al_5_O_12_.

Thermal barrier materials need to possess a coefficient of thermal expansion similar to that of a high-temperature alloy matrix because a thermal expansion mismatch can cause cracking of the matrix. Figure 7 shows the curves of the linear thermal expansion of (Y_0.2_Dy_0.2_Ho_0.2_Er_0.2_Yb_0.2_)_3_Al_5_O_12,_ determined from room temperature to 1273 K. The relative changes in the length of the (Y_0.2_Dy_0.2_Ho_0.2_Er_0.2_Yb_0.2_)_3_Al_5_O_12_ samples were recorded with the increasing temperature.

In addition, the (Y_0.2_Dy_0.2_Ho_0.2_Er_0.2_Yb_0.2_)_3_Al_5_O_12_ was designed using a pressureless sintering method, with a coefficient of thermal expansion of 9.28 × 10^−6^ K^−1^ at 673–1273 K, approximately 18.52% higher than that of Yb_3_Al_5_O_12_ (7.83 × 10^−6^ K^−1^, 673–1273 K), and approximately 8.67% higher than that of (Y_0.2_Yb_0.2_Lu_0.2_Eu_0.2_Er_0.2_)_3_Al_5_O_12_ (8.54 × 10^−6^ K^−1^, 673–1273 K); thus, this material can be applied to various substrates, such as typical high-temperature nickel-based alloys [40].

**Figure 8:**
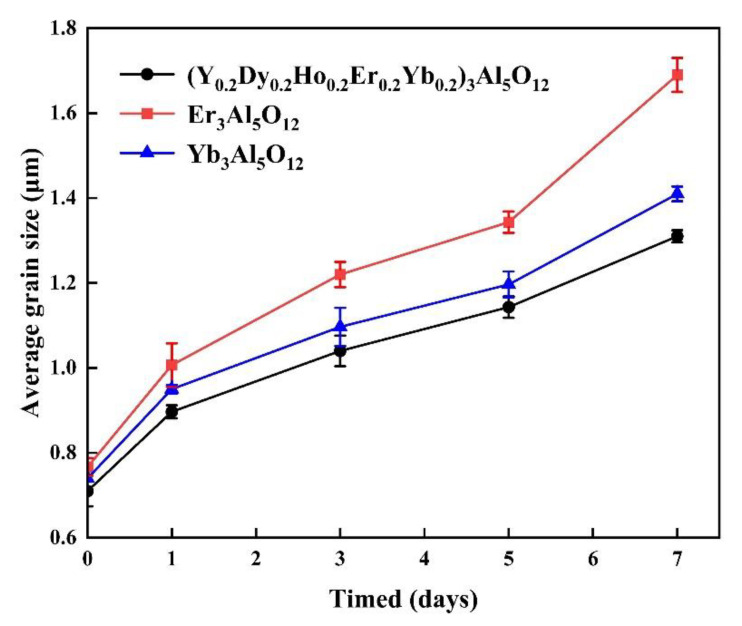
Average grain size of (Y_0.2_Dy_0.2_Ho_0.2_Er_0.2_Yb_0.2_)_3_Al_5_O_12_, Er_3_Al_5_O_12,_ and Yb_3_Al_5_O_12_ ceramics after annealing at 1550 °C for 7 days in air.

A low grain growth rate is very important for TBC materials, as it improves the crack resistance due to thermal stress and prevents an increase in thermal conductivity. Figure 8 shows the average grain sizes of the (Y_0.2_Dy_0.2_Ho_0.2_Er_0.2_Yb_0.2_)_3_Al_5_O_12_ ceramic specimens after annealing at 1550 °C for 7 days. As seen in Figure 8, the (Y_0.2_Dy_0.2_Ho_0.2_Er_0.2_Yb_0.2_)_3_Al_5_O_12_ ceramics have a lower average grain rate than the Yb_3_Al_5_O_12_ and Er_3_Al_5_O_12_. The grain growth rates of the (Y_0.2_Dy_0.2_Ho_0.2_Er_0.2_Yb_0.2_)_3_Al_5_O_12_, Yb_3_Al_5_O_12_, and Er_3_Al_5_O_12_ samples were similar during the annealing period of 0-5 days at 1550 °C. However, when the annealing time was extended to 7 days, the grain growth rate of the high-entropy aluminate was less than that of the Yb_3_Al_5_O_12_ and Er_3_Al_5_O_12_ under the same conditions. As a result of sluggish diffusion in HECs, (Y_0.2_Dy_0.2_Ho_0.2_Er_0.2_Yb_0.2_)_3_Al_5_O_12_ ceramics grow slowly because fine particles are retained under high temperatures [39].

**Figure 9:**
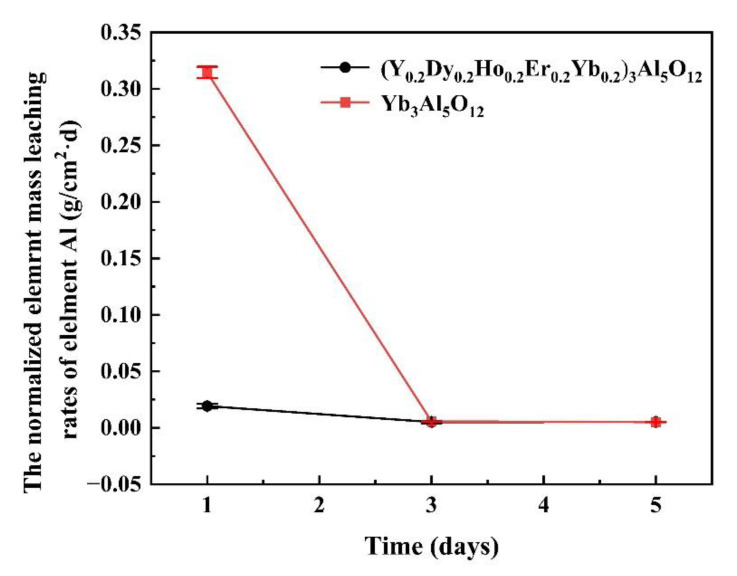
Normalized elemental leaching rates of Al in (Y_0.2_Dy_0.2_Ho_0.2_Er_0.2_Yb_0.2_)_3_Al_5_O_12_ and Yb_3_Al_5_O_12_ for 5 days.

Thermal barrier materials must have excellent chemical inertness. Figure 9 shows the normalized elemental leaching rates of Al in (Y_0.2_Dy_0.2_Ho_0.2_Er_0.2_Yb_0.2_)_3_Al_5_O_12_ and Yb_3_Al_5_O_12_ over 5 days. The elemental aluminum leaching rate of the (Y_0.2_Dy_0.2_Ho_0.2_Er_0.2_Yb_0.2_)_3_Al_5_O_12_ remained low during 1–5 days of corrosion in an acidic environment. However, the single-component garnet-structured aluminate ceramics exhibited a large leaching rate of elemental aluminum. This result indicated that (Y_0.2_Dy_0.2_Ho_0.2_Er_0.2_Yb_0.2_)_3_Al_5_O_12_ ceramics have better chemical stability than Yb3Al5O12. Therefore, high-entropy aluminate ceramics are promising materials for TBCs.

**Figure 10:**
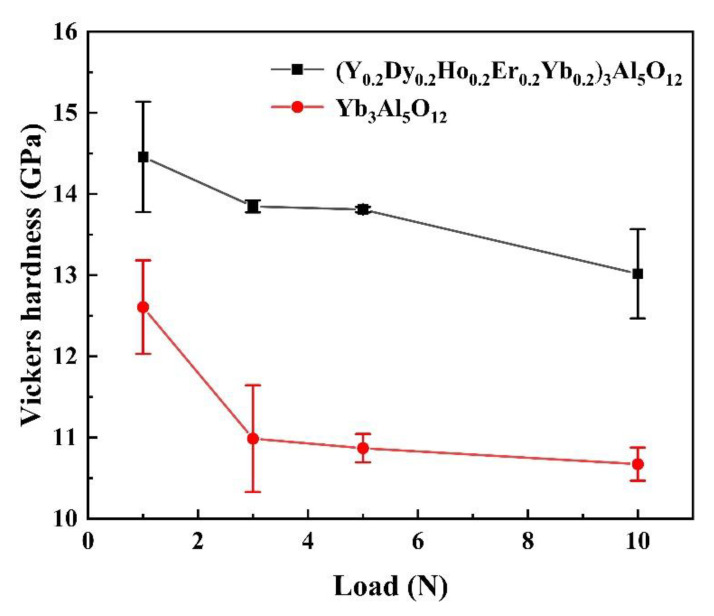
Vickers hardness of (Y_0.2_Dy_0.2_Ho_0.2_Er_0.2_Yb_0.2_)_3_Al_5_O_12_ and Yb_3_Al_5_O_12_ versus load.

The mechanical property of a TBC is also important as mechanical damage such as impact or wear can apply to the TBC. The mechanical property data of (Y_0.2_Dy_0.2_Ho_0.2_Er_0.2_Yb_0.2_)_3_Al_5_O_12_ are also listed in Figure 10 as a comparison. As can be seen in Figure 10, the hardness of (Y_0.2_Dy_0.2_Ho_0.2_Er_0.2_Yb_0.2_)_3_Al_5_O_12_ is significantly higher than that of Yb_3_Al_5_O_12_. The hardness of (Y_0.2_Dy_0.2_Ho_0.2_Er_0.2_Yb_0.2_)_3_Al_5_O_12_ is determined to be 13.3 GPa, which is higher than that of Yb_3_Al_5_O_12_ (10.7 GPa), and slightly lower than YSZ (14.0 GPa). The excellent mechanical property data suggest that (Y_0.2_Dy_0.2_Ho_0.2_Er_0.2_Yb_0.2_)_3_Al_5_O_12_ can be used as a potential thermal insulation material.

## 4. Conclusions

In summary, high-entropy rare-earth aluminate thermal barrier materials with garnet structures, (Y_0.2_Dy_0.2_Ho_0.2_Er_0.2_Yb_0.2_)_3_Al_5_O_12_ ceramics, were prepared by a solid-state reaction method. The results of XRD, SEM, and TEM analysis show that the synthesized (Y_0.2_Dy_0.2_Ho_0.2_Er_0.2_Yb_0.2_)_3_Al_5_O_12_ ceramic powder consists of a pure phase with a homogeneous rare-earth element distribution, and the lattice dimensions are *a* = *b* = *c* = 11.992 Å. According to the refined dimensions of the cell, the theoretical density of the (Y_0.2_Dy_0.2_Ho_0.2_Er_0.2_Yb_0.2_)_3_Al_5_O_12_ was calculated to be 6.02 g/cm^3^. The sintered density calculated by Archimedes’ approach is 5.88 g/cm^3^, which is 97.67% of the theoretical value. The thermal conductivity of (Y_0.2_Dy_0.2_Ho_0.2_Er_0.2_Yb_0.2_)_3_Al_5_O_12_ ceramics is 3.48 W/(m·K) at 300 K, approximately 25.48% and 8.66% lower than that of Yb_3_Al_5_O_12_ (4.67 W/(m·K)) and (Y_0.2_Yb_0.2_Lu_0.2_Eu_0.2_Er_0.2_)_3_Al_5_O_12_ (3.81 W/(m·K)). The thermal expansion of (Y_0.2_Dy_0.2_Ho_0.2_Er_0.2_Yb_0.2_)_3_Al_5_O_12_ is (9.28 × 10^−6^ K^−1^, 673-1273 K)), approximately 18.52% higher than that of Yb3Al5O12 (7.83 × 10^−6^ K^−1^, 673–1273 K) and approximately 8.67% higher than that of (Y_0.2_Yb_0.2_Lu_0.2_Eu_0.2_Er_0.2_)_3_Al_5_O_12_ (8.54 × 10^−6^ K^−1^, 673–1273 K). After annealing the (Y_0.2_Dy_0.2_Ho_0.2_Er_0.2_Yb_0.2_)_3_Al_5_O_12_ at 1550 °C, its average grain size was found to be lower than that of Yb_3_Al_5_O_12_ and Er_3_Al_5_O_12_, due to the sluggish diffusion effects. As the annealing time was extended to 7 days, the average grain size of the high-entropy aluminate became much smaller than that of the Yb_3_Al_5_O_12_ and Er_3_Al_5_O_12_ under the same conditions. In addition, (Y_0.2_Dy_0.2_Ho_0.2_Er_0.2_Yb_0.2_)_3_Al_5_O_12_ has excellent chemical stability compared with Yb_3_Al_5_O_12_. The results show that (Y_0.2_Dy_0.2_Ho_0.2_Er_0.2_Yb_0.2_)_3_Al_5_O_12_, in comparison with Yb_3_Al_5_O_12_, is a potential thermal barrier material.

## Figures and Tables

**Table 1:** Refined cell size and theoretical density of as-synthesized (Y_0.2_Dy_0.2_Ho_0.2_Er_0.2_Yb_0.2_)_3_Al_5_O_12_ and the five single component garnets.

Compounds	Theoretical Density (g/cm^3^)	Relative Density	*a* (Å)
(Y_0.2_Dy_0.2_Ho_0.2_Er_0.2_Yb_0.2_)_3_Al_5_O_12_	6.02	97.70%	11.992
Y_3_Al_5_O_12_	4.56	-	12.000
Dy_3_Al_5_O_12_	6.20	-	12.038
Ho_3_Al_5_O_12_	6.32	-	12.000
Er_3_Al_5_O_12_	6.43	-	11.962
Yb_3_Al_5_O_12_	6.62	-	11.930

**Table 2:** The thermal conductivity of the (Y_0.2_Dy_0.2_Ho_0.2_Er_0.2_Yb_0.2_)_3_Al_5_O_12_ and Y_3_Al_5_O_12_ at 300 K.

	(Y_0.2_Dy_0.2_Ho_0.2_Er_0.2_Yb_0.2_)_3_Al_5_O_12_	Yb_3_Al_5_O_12_	YSZ
Thermal conductivity (W/(m·K))	3.48	4.67	2.50–3.20

## Data Availability

The data presented in this study are available from the corresponding authors upon reasonable request.
